# Supplementary material for: Cost-aware orchestration of applications over heterogeneous clouds
Source: PLoS One. 2020 Feb 18;15(2):e0228086. doi: 10.1371/journal.pone.0228086 (PMC7028274; doi:10.1371/journal.pone.0228086)
Supplement: Attachment — Submitted filename: PONE 2019 Response Letter V1.7.pdf [file pone.0228086.s003.pdf]

PONE-D-19-20789

Cost-Aware Orchestration of Applications over Heterogeneous Clouds

The authors would like to acknowledge that we have done our best to revise the manuscript according to the comments and suggestions raised by the reviewers. We are very grateful to the anonymous reviewers for their comments, which we found helpful and enlightening. We would also like to point out that we have addressed the comments by the editor as well. The responses to those comments are highlighted in the revised manuscript.

We believe that the manuscript is now in better shape in many aspects, including a clearer description of our proposed system and improved presentation of figures and tables. The proposed scheme has also been improved to be able to consider burstable instances when making orchestration decisions.

Editor Comments to the Author

Thank you for updating your data availability statement. You note that your data are available within the Supporting Information files, but no such files have been included with your submission. At this time we ask that you please upload your minimal data set as a Supporting Information file, or to a public repository such as Figshare or Dryad.

Please also ensure that when you upload your file you include separate captions for your supplementary files at the end of your manuscript.

As soon as you confirm the location of the data underlying your findings, we will be able to proceed with the review of your submission.

In response to this comment, we explained in the manuscript that the experimentations were carried out using the simulation data. We also include the raw data and our code in the supplementary files with the submission of the updated version of the manuscript.

Please explain the rationale for the development of your method in light of recent research in this area, clearly indicating which problem with existing methods you are addressing.

Please clearly report at the beginning of your methods or results section which were the key performance measures used to establish the validity and utility of your method. Please also report clearly which statistical analysis was used to establish robustness of performance measures.

In response, we updated the first paragraph of the "Evaluation of Results" section and included the cost to detect the cost violation as well as the approach we used to scale with multiple services. The modified part of the section is as follows.

Evaluation of Results -- Modified Part

"In evaluating our work, we chose to perform a policy enforcement performance evaluation and a cost-awareness evaluation. For our policy enforcement performance evaluation, it was important that we measure key areas of performance of our approach. For this, we chose a two point approach,

- *Determine the time to detect a cost violation*
- *Determine how our approach scales with multiple services*

In determining the time it takes to detect and initiate an action for a cost violation, it was clear that our approach needed to be able to detect a violation within the smallest billing period used by cloud service providers. A majority of cloud service providers make use of per-second billing, hence, it was important that we determine whether our approach can detect a violation in time less than one (1) second. Once we established if our approach can feasibly detect a violation within an appropriate time, it was important that we determine how our approach scales to applications comprising more than one node. As most microservice applications are represented by multiple, interconnected service, then our approach needed to scale such that applications of more than one node also provided detection times of less than one (1) second. For our cost-awareness evaluation, we chose to determine how our approach reacts to various scenarios. It was important that our approach be capable of not only detecting a violation but also determining the appropriate action in each scenario."

-------------**-----------*****-----------------****---------***-------****-----------**

Furthermore, we would like to acknowledge that all the other minor comments by the editor are also taken care of including validation of ORCiD validation, attaching .tex file of the manuscript, removing acknowledgment section from the manuscript, and updating our funding statement accordingly.

<<--->>

Review Comments to the Author

Reviewer #1: Thanks for submitting your work! The paper overall is well written and describes a way to do cost-aware orchestration on an application component level. This is an important part of orchestration and worthy to be published.

The results look promising, albeit a bit incremental to related work - as also pointed out by the authors in the related work section. However I belief that with some minor revisions this work could be set apart from what has been done to date and show a greater impact. I'd suggest to look at dynamic pricing e.g. burstable instances in public Clouds like AWS. For example by knowing that some components of the application have low average CPU utilization but have occasionally utilization spikes - further cost saving could be achieved. This dynamicity would be a nice addition to the work presented in the paper - For initial reference see: https://docs.aws.amazon.com/AWSEC2/latest/UserGuide/burstable-performance-instances-unlimited-mode.html

Hope the authors consider this recommendation - and I wish them the best for their submission!

In response to this comment, we looked into the burstable instance model offered by AWS and incorporated burstable instance types into our algorithm and other related sections as advised. We modified our policy management, cost-aware policy manager, cost orchestration scheme, and cost orchestration algorithm. New evaluation results are added using burstable instances in the revised manuscript.

We have introduced the BURST_ABOVE action with the previously defined REFACTOR action, which handles the situations where the budget may change because of a momentary surge in the CPU demand of the specified VM. The modified part of the section is as follows.

Policy Management -- Modified Part

"The REFACTOR action, however, can also affect a change in other properties. Since these other properties are not part of our policy, the policy manager need not worry about their changes and may still choose to carry out a REFACTOR action. Conversely, a budget change may be indirectly caused by an increase in CPU performance of the VM. In this case, it is possible to affect the operating cost through the use of a REFACTOR or BURST_ABOVE action, if the current VM is burstable. It should be noted that policy directives serve to instruct how an attribute is expected to change, after an action is carried out. However, the algorithmic logic used in the selection of the directives is built into the policy manager."

-------------****----------*****-----------------****---------***-------****-----------

We also included an example of using burstable instances for an application component that allows the policy manager to burst above the baseline utilization in case of any violations to the performance of the application. Therefore, in the event of performance violations, our policy manager turns to the proposed orchestration scheme to shape the appropriate action to be carried out. The modified part of the paragraph is as follows.

Cost-Aware Policy Manager -- Modified Part

"For example, if an application component is deployed using a burstable VM, then the policy manager should be aware of the option to burst above the baseline utilization in the event of a performance violation. Our policy manager is also afforded all the necessary information about the running application components, e.g., the location and tier of the deployed service as well as the configuration of the VM used to fulfil it. Consider an application component deployed using a VM based on Microsoft Azure's general-purpose category using an A0 tiered VM. This image gives 1 CPU core and is priced at a rate of $0.018 per hour on demand as given in Table 1. The cost of 2 CPUs in this tier, however, jumps to $0.068 per hour. To make use of this information, our cost-aware policy manager utilizes a cost orchestration algorithm as well as a service catalog that allows it to query the rate of services offered by different providers. Based on the actions that are available to the policy manager supplied as policy management directives, the policy manager uses our cost orchestration scheme to determine the actions to be carried out."

-------------****----------*****-----------------****---------***-------****-----------

Furthermore, we defined the BURST_ABOVE action in our cost orchestration scheme that allows the system to respond to performance fluctuations for burstable instances. This BURST_ABOVE action is an alternative to the REFACTOR action previously defined as an appropriate action for the violation of the

cost constraint of the application. The orchestration algorithm now considers the BURST_ABOVE action in situations that allows increasing the CPU performance of current VMs instead of triggering VM changes. Some of the additional paragraphs and modified figures added to the revised manuscript are as follows.

Cost Orchestration Scheme -- Modified Part

"Our cost orchestration algorithm is critical to determining the action as well as the carrying them out. Looking at our policy management directives, we have defined a REFACTOR action as an appropriate action to be carried out in the case of a violation of a cost constraint. Considering that a performance violation may also trigger a change in the application's topology, then it is also important that we define a BURST ABOVE action as well. The definitions are shown in Fig. 5. In the event that the policy manager decides that a REFACTOR or action is suitable to solve the issue, it is important that we properly evaluate them to ensure that, when executed, results in a favorable solution. If we consider a scenario where we are required to reduce the operating cost of a service, we can identify the following actions:

1. *Move (REFACTOR) the service to another VM on the same tier with a lower CPU core count.*
2. *Move (REFACTOR) the service to another VM on a different tier of the same provider, with or without lowering the CPU core count.*
3. *Move (REFACTOR) the service to another VM on another provider with or without lowering the CPU core count.*

In the scenario where an increase in performance would necessitate a VM in a higher tier, which would incur a higher cost then in order to maintain the current operating cost, the following actions may be considered.

4. *Increase (BURST_ABOVE) the CPU performance of the current VM to meet the performance needs rather than triggering a change of VM.*

```
- id: REFACTOR
   actions:
   - property: PROVISIONING_LOCATION
      transitions:
      - type: Initial
         value: ANYTHING
      - type: Change
         value ANYTHING
    - property: SERVICE_COST
      transitions:
      - type: Initial
         value: ANYTHING
      - type: Change
         value ANYTHING
   - property: APPLICATION_COST
      transitions:
      - type: Initial
         value: ANYTHING
      - type: Change
         value ANYTHING
   - property: EXPECTED_APPLICATION_COST_PER_YEAR
      transitions:
      - type: Initial
         value: ANYTHING
      - type: Change
         value ANYTHING
- id: BURST_ABOVE
   actions:
   - property: CPU_CREDIT
      transitions:
      - type: Initial
         value: ANYTHING
      - type: DecreaseBy
         value: CPU_CREDIT_RATE"
```

Fig 5. Refactor policy directive.

Our policy manager must therefore be equipped with an algorithm to determine how to move the service in order to mitigate the cost constraint violation. The outline of the algorithm used in our work is given in Fig. 6. Here we used a greedy algorithm to test the responses of the responses of the policy manager to ensure that it evaluated the directives it was given. The algorithm contains modules for refactoring as well as bursting. Depending on the scenario, either may be chosen as a viable option to rectify a policy violation. The module will loop until the service is refactored or it exits due to there being no viable option to refactor to. The refactor sub-module finds three minimum value services that may resolve the violation: the smallest service on the same tier on the same provider, the smallest service of another tier on the same provider, and the smallest service of any tier on another provider. The minimum of these three is the smallest service that may resolve the violation. In the case of a performance violation, a burst action will always be considered over performing a refactor action, as this action does not alter the operating cost."

```
public void refactorDown(BUDGET) {
   exit = false;
   while(!exit){
      if (application.violation(BUDGET)){
         refactored = false;
         sortedServices = sortDescending(services);
         for each Service svc in sortedServices {
            current = getCurrentProvider(svc);
            refactored = (!performRefactor(svc).equalsTo(current) || false );
            if (!application.violates(BUDGET)) {
               exit = true;
            }
         }
         if ( !refactored ) exit = true;
      }  else
      exit = true
   }
}

public void refactorUp(BUDGET){
   exit = false;
   while(!exit){
      if (application.violation(BUDGET)){
         refactored = false;
         sortedServices = sortAscending(services);
         for each Service svc in sortedServices {
            current = getCurrentProvider(svc);
            refactored = (!performRefactor(svc).equalsTo(current) || false );
            if (!application.violates(BUDGET)) {
               exit = true;
            }
         }
         if ( !refactored ) exit = true;
      }  else
      exit = true
   }
}

performRefactor(Service svc, Catalog stream) {
   Provider min1 = stream.filter(svc.location, svc.tier)
   .min(Comparator.comparing(Service::getRate()))
   .orElse(DEF_RATE);
   Provider min2 = stream.filter(svc.location, svc.tier)
   .min(Comparator.comparing(Service::getRate()))
   .orElse(DEF_RATE);
   Provider min2 = stream.filter(svc.location, svc.tier)
   .min(Comparator.comparing(Service::getRate()))
   .orElse(DEF_RATE);

   return MinProvider(min1, min2, min3)
}

performBurst(Service svc) {
   final ScheduledExecutorService executorService =
   Executors.newSingleThreadScheduledExecutor();
   executorService.scheduleAtFixedRate(new Runnable() {
      @Override
      public void run() {
         if (svc.cpuCredit() <= 0 || svc.cpuUtilization() <= svc.BASELINE) return;
      }
   }, 0, 1, TimeUnit.SECONDS);
}
```

Fig 6. Cost orchestration algorithm.

___________****___________*****________________****_________***_______****___________

Furthermore, we also present the discussion of the analytical results by inserting additional explanation, analysis, and supporting data in the revised version of the manuscript to address the comments. With the addition of the burstable instances, there is a need to determine the time taken to evaluate a REFACTOR or BURST_ABOVE decision for a single service in cases where its cost constraints get violated. Also, an additional orchestration scenario is devised where the system responds to increase in the CPU utilization above baseline as shown in the modified version of Table 4. Some of the modified results and explanation added to the revised manuscript are as follows.

Policy Enforcement Performance – Modified Part

"First, we need to determine the time taken to evaluate a REFACTOR or BURST_ABOVE decision for a single service, when its cost constraint gets violated. To do this, we created a deployment with a single service, triggered a cost violation on that service, and recorded the time required to evaluate the decision. The results were tabulated and the experiment was then repeated. The tests were run for approximately 5000 iterations, after which we were able to determine the average time to detect and respond to a cost constraint violation. This was found to be approximately 0.9846ms. Fig. 7 shows the results of this baseline test."

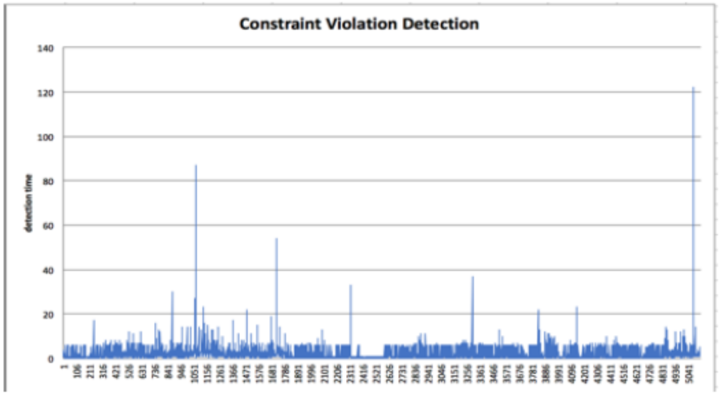

Fig 7. Baseline conversion times.

Cost-Awareness Evaluation -- Modified Part

"In our scenarios, we treat budgeted cost as a range allowing us to manipulate the lower and upper bounds of the range. The scenarios explored are as follows:

- *Response to increase in upper bounds of Budget.*
- *Response to decrease in upper bounds of Budget.*
- *Response to increase in lower bounds of Budget.*
- *Response to decrease in lower bounds of Budget.*
- *Response to Increase in CPU utilization above Baseline.*

Prior to beginning the tests, we configured the application with an initial cost control policy which specifies that our application's cost should be maintained between the range of $300 and $500. Once specified, we deployed the application components across the cloud providers. The initial component distribution and

configuration is given in Table 3. We then ran each scenario and tabulated the results obtained from our system which we present in Table 4. We can see that, in the first scenario, if the upper limit of the budget is increased to $600, then the system responds with no change. In scenario two, the upper budget constraint is decreased to $350, which caused the system to respond by refactoring comp 1 and comp 2.The refactor actions resulted in the service being moved to VMs of the same flavours in the different region. When the lower budget was reduced, however, no refactoring was recorded. Also, no refactoring was done for scenarios 1 and 4, as these scenarios would not result in a policy violation. Finally in the last scenario, when the CPU utilization was increased, rather than performing a refactor to another VM, the policy manager instead performed a burst action which cause the budget to remain unchanged."

Table 4. Response to budget constrain changes and CPU utilization

Scenario		Action	Final					
				l	i	c	m	Rate
1. Upper Budget Increase		No Action	comp 1	AWS Tokyo	t2.large	2	8	$0.13
$300.00	$600.00	No Action	comp 2	AWS Sydney	t2.large	2	8	$0.13
		No Action	comp 3	Rackspace HK	General 1-4	4	4	$0.15
		No Action	comp 4	Rackspace SYD	General 1-4	4	4	$0.15
					Projected Operating Cost			$397.44
2. Upper Budget decrease		Refactor	comp 1	AWS Virginia	t2.large	2	8	0.094
$300.00	$350.00	Refactor	comp 2	AWS Virginia	t2.large	2	8	0.094
		No Action	comp 3	Rackspace HK	General 1-4	4	4	0.148
		No Action	comp 4	Rackspace SYD	General 1-4	4	4	0.148
					Projected Operating Cost			$348.48
3. Lower Budget Increase		Refactor	comp 1	AWS Tokyo	m3.large	2	7.5	$0.19
$450.00	$500.00	Refactor	comp 2	AWS Sydney	m3.large	2	7.5	$0.19
		No Action	comp 3	Rackspace HK	General 1-4	4	4	$0.15
		No Action	comp 4	Rackspace SYD	General 1-4	4	4	$0.15
					Projected Operating Cost			$486.00
4. Lower Budget decrease		No Action	comp 1	AWS Tokyo	t2.large	2	8	$0.13
$200.00	$500.00	No Action	comp 2	AWS Sydney	t2.large	2	8	$0.13
		No Action	comp 3	Rackspace HK	General 1-4	4	4	$0.15
		No Action	comp 4	Rackspace SYD	General 1-4	4	4	$0.15
					Projected Operating Cost			$397.44
5. Increase CPU utilization								
15%	65%	Burst_Above	comp 1	AWS Tokyo	t2.large	2	8	$0.13
15%	65%	Burst_Above	comp 2	AWS Sydney	t2.large	2	8	$0.13
		No Action	comp 3	Rackspace HK	General 1-4	4	4	$0.15
		No Action	comp 4	Rackspace SYD	General 1-4	4	4	$0.15
					Projected Operating Cost			$397.44

References

1. C. Wang, B. Urgaonkar, N. Nasiriani, and G. Kesidis, "Using Burstable Instances in the Public Cloud: Why, When and How?," in Proc. ACM SIGMETRICS Int. Conf. Measurement and Modeling of Computer Systems, Urbana-Champaign, IL, USA, 2017, pp.56-56.

<<--->>

Reviewer #2: This work focused on the orchestration strategy to manage the cost of applications over clouds. The authors considered a scenario of microservice applications to illustrate their approach. After reviewing this paper, couples of comments are provided below.

1) In the motivation and challenges part, the authors illustrated the motivation to control the cost of an application by different configurations of VMs. But the importance of the cost-aware orchestration has not been addressed. Before talking about challenges in the topology model, cost model, and policy model respectively, the relationship among these three models should be explained clearly. And then what the problems and difficulties in designing can be illustrated.

Cloud applications are usually subjected to varying conditions during the application or its components lifetime. These varying conditions could be spikes in utilization, base cost changes of VMs that are part of the deployment, or maybe removal or addition of any flavors of VM from the service provider catalog. Therefore, applications need to be aware of the changes in operational costs over time and have the capability of responding appropriately. In response to this comment, we modified our motivation and challenges section. The modified part of the section in the revised manuscript reads as follows.

Motivation and Challenges -- Modified Part

"In looking at the motivation for our study firstly, we considered the fact that a cloud provider provides virtual machine (VM) images based on different tiers, according to the hardware configuration of the servers required. As each tier of VM utilizes a varying amount of resources from the provider's infrastructure, it is only fitting that these tiers be priced differently based on the resources used in the provider's datacenter. Microsoft Azure, for example, categorizes its services based on use cases such as General Purpose, Compute Optimized, Memory Optimized, Storage Optimized, GPU and High Performance Compute [10]. Each of these is further subdivided. For example, General Purpose category is broken into B, A, and D tiers, as shown in Table 1. Amazon's AWS on the other hand provides a slightly different scheme for their VMs by dividing into both a kind (General, Compute, etc.) and a flavor. Table 2 gives us a look at a subsection of AWS's VM classification and division [11]. Apart from being subdivided based on the configuration of the VMs, cloud service providers have also started providing VMs that are burstable such that their performance can burst above a given average and not incur added cost [12]. These VM's offer a baseline performance at a significantly lower cost to traditional VMs.

Importantly, what can be seen is that VMs are varied based on different metrics such as the number of cores and the amount of RAM that is available. Considering that an application may utilize varying numbers of VMs, each with different configurations, the operating cost of the application should be related to the configuration of VMs used to deploy the application. Applications deployed to cloud service providers' infrastructure are usually subjected to varying conditions during their lifetime. Applications may be subjected to spikes in utilization, changes in the base cost of VMs used in their deployments or the addition or removal of flavors of VMs from the catalog of VMs offered by providers. As such any one of these situations may result in changes in the cost to operate the application over time. It is therefore important that an application be aware of these changes and be capable of responding appropriately in

order to mitigate their effects. Our work is, therefore, based on providing an orchestration strategy capable of managing the cost of an application by controlling the configuration of the VMs used to compose the application. To illustrate our strategy, we consider a situation involving a microservice application. The application comprises a front-end written in angular TS and running on Node.js as well as three back-end components, i.e., users, products, and orders. Each back-end component is a Java application that runs on an embedded Jetty server and uses its own Mongo DB data store."

2) In the Cost Model for Cloud Orchestration, the model is defined in the component level. The reason for the cost model is independent of cloud providers platforms should be explained. And which parts make your approach application-aware need to be clarified.

In response, we modified our cost model section by adding details of how the cost model is independent of the cloud provider. In cloud applications, a service can be fulfilled through multiple means such as VMs, containers, and so on. Therefore, the proposed cost model must be independent of the vendor-specific technology, leading to situations where different providers can fulfill the service need without any cost dependence. This model also works in multi-cloud situations where equivalent services can be acquired from multiple service providers. Our proposed solution makes it possible by making the cost model as a function of common factors of VMs used to fulfill services, and then the model is embedded into policy orchestrator to be combined with the application topology and policies. The modified part of the cost model section in the revised manuscript is as follows.

Cost Model -- Modified Part

"Application blueprints deployed to the cloud may be fulfilled in different ways depending on the technology requested by the customer or employed by the vendor. For example, an application deployed to Kubernetes is fulfilled using Docker containers clustered to form logical "pods", whereas an application deployed to Amazon's EC2 service may be fulfilled using virtual machine instances [13-15]. As it is possible for a service to be fulfilled through various means, it is important that the model used to define the cost of an application be independent of vendor specific technology. Hence, the cost accrued by a service deployed on one provider remains unaffected in the event that another provider must fulfill that service. Not only is it important that the model be vendor agnostic in order to allow cost to be accumulated regardless of the method used to fulfill the service, the model should also allow for the accumulation of cost for services deployed across multiple providers. This is usually the case with multi-cloud applications. To capture these properties, we chose to express the cost model as a function of the common factors of VMs used to fulfill services in provider clouds, then translate that function into an algorithmic form, embedding it into our policy orchestrator so that it may be combined with the topology and policies that define our application."

3) The authors did a good study in the related work for TOSCA and other external cost control services. A minor error should be noted. ...[?, 20, 26]... in line #427.

The error has been corrected in the revision as shown below.

"In addition to the aforementioned approaches, there have been various work dedicated to cost-aware scaling of cloud applications [25-28]. A cost-aware system, named Kingfisher solution, was presented that considers other elasticity mechanisms such as replication and migration [25]."

4) Figure 4,5,6 and table 1,2 look like a screenshot. Please try to use latex or other utilities to generate table or algorithm blocks to make them clear enough when zooming in.

In response to this comment, we regenerate the figures and tables using latex. We have confirmed that the modified version of the tables and figures are now clear enough when zoomed in the enhanced version of the manuscript.
